# Pandemic Effects and Gluten-Free Diet: An Adherence and Mental Health Problem

**DOI:** 10.3390/nu13061822

**Published:** 2021-05-27

**Authors:** Karla A. Bascuñán, Juan Manuel Rodríguez, Carla Osben, Alan Fernández, Carlos Sepúlveda, Magdalena Araya

**Affiliations:** 1 Institute of Nutrition and Food Technology (INTA), University of Chile, Santiago 7810000, Chile; karlabascunan@gmail.com (K.A.B.); Juan.rodriguez@inta.uchile.cl (J.M.R.); carla.osben@gmail.com (C.O.); alfernandezbe@gmail.com (A.F.); csepulvedag@ug.uchile.cl (C.S.); 2 Department of Nutrition, Faculty of Medicine, University of Chile, Santiago 8380453, Chile; 3 Laboratorio de Ciencias del Ejercicio, Clínica Meds, Santiago 7500000, Chile; 4 Corporación de Apoyo al Celíaco (COACEL), Santiago 7810000, Chile

**Keywords:** gluten-related disorders, celiac disease, pandemic, adherence, anxiety, depression

## Abstract

The COVID-19 pandemic has been present for many months, influencing diets such as the gluten-free diet (GFD), which implies daily challenges even in non-pandemic conditions. Persons following the GFD were invited to answer online ad hoc and validated questionnaires characterizing self-perceptions of the pandemic, current clinical condition, dietary characteristics, adherence to GFD, anxiety, and depression. Of 331 participants, 87% experienced shortage and higher cost of food and 14.8% lost their jobs. Symptoms increased in 29% and 36.6% failed to obtain medical help. Although 52.3% increased food preparation at home and purchased alternative foodstuffs, 53.8% had consumed gluten-containing foods. The Health Eating Index was intermediate/“needs improvement” (mean 65.6 ± 13.3 points); in 49.9% (perception) and 44.4% (questionnaire), adherence was “bad”. Anxiety and depression scores were above the cutoff in 28% and 40.4%, respectively. Adherence and mental health were strongly related. The likelihood of poor adherence was 2.3 times higher (*p* < 0.004) in participants declaring that pandemic altered GFD. Those suffering depressive symptoms were 1.3 times more likely to have poor adherence (*p* < 0.000). Depression and faulty GFD (mandatory for treatment) appear, affecting a high proportion of participants, suggesting that support measures aimed at these aspects would help improve the health condition of people that maintain GFD. Comparisons of data currently appearing in the literature available should be cautious because not only cultural aspects but conditions and timing of data collection are most variable.

## 1. Introduction

In December 2019, the first cases of SARS-Cov2 in humans were detected in Wuhan, China [1]. The virus triggered an epidemic that rapidly spread globally as an acute respiratory syndrome [2]; involvement of gastrointestinal and other systems was subsequently reported [3]. The WHO declared a pandemic in March 2020 [4]. Massive worldwide lockdowns and quarantines, and other measures such as physical distancing and self-isolation ensued to protect life and avoid health systems collapse. In Chile, dynamic quarantines were initiated in March and lockdown began in April 2020. Due to the enormous number of COVID-19 cases, ambulatory and follow-up consultations for chronic diseases were usually suspended. All these strategies produced sudden and radical changes in people habits and lifestyles, drastically reducing social interaction, affecting everyday behaviors, and eating habits, among other things.

Today, celiac disease (CD) and other chronic diseases that require treatment with specific restrictive diets raise concern among specialists. The first descriptions of pandemic effects appeared early; in May 2020, Siniscalchi et al. [5] reported the acute effects identified in 276 adult celiac patients. They did not feel more vulnerable due to their condition, and they did not worry much about the possible shortness of gluten-free food during the epidemic (“not at all” = 48.5%). The most worried were elderly patients, those suffering comorbidities, and females [5]. At the same time, depression and anxiety were reported at 27.9% and 31.6%, respectively, in China [6]; Kontoangelos et al. reviewed the psychological effects of the COVID-19 pandemic, describing that children, older people, and those with underlying health conditions are likely to feel worry, anxiety, and fear, which can be extremely frightening [7].

After experiencing the pandemic effects throughout 2020, we are now learning about the consequences that long isolation measures had on people; indeed, clinical status of diseases, access to food, and mental health are most relevant in patients with gluten-related disorders (GRD) that must keep treatment with gluten-free diet (GFD). Maintaining this diet is always challenging, and patients often feel worried and isolated [8]. GFD is the only known treatment for these conditions [9] and although effective, it may be deficient in macro and/or micronutrients and vitamins, low in fiber, and may have high glycemic index and other shortcomings [7]. Gluten-free foods are typically less available than those forming the regular gluten-containing diet and may be three or more times more expensive than gluten containing equivalents [10]. With the hypothesis that after living for many months in the conditions imposed by the pandemic, the effects on GFD are pronounced and detectable, we invited persons following this diet (due to CD, non-celiac gluten sensitivity, wheat allergy, or GFD as an option of healthy diet) to answer an online questionnaire that characterized four aspects: perceptions about the general pandemic effects, current clinical conditions and dietary characterization, adherence to GFD, and mental health (anxiety and depression).

## 2. Material and Methods

This population-based, cross-sectional online study was conducted in October–November 2020. An ad hoc questionnaire specially developed for this study asked for sociodemographic characteristics, the perceived effects of the pandemic proper (worrying for risk of infection, shortage of foods, isolation, need to go out of home), dietary data (reasons for maintaining GFD, years on GFD, self-perception of adherence, effects of pandemic on diet, access to gluten-free foods), frequency of home cooking, ingredients/foods the replaced those unavailable, weekly frequency consumption of gluten-containing foods, cost of gluten-free foods, clinical data (diagnosis if any, year of diagnosis, symptoms during last four months, need to consult during last 4–6 months, perceived weight changes), diagnosis of anxiety or depression disorders before or during the pandemic. Validated questionnaires were applied for dietary data, adherence, and mental health: (i) *Healthy eating index* (HEI) [11]; ten variables that define dietary nutritional quality and yield a maximum of 100 points, classifying results into “healthy” (>80), “needs improvement” (51–80), and “not healthy” (≤50). (ii) *Celiac disease adherence test* (CDAT) [12,13]; 7-item scale that classifies responders into “adherent” (patients scoring 7–12 points) and “non-adherents” (scores ≥ 13 points) to GFD. (iii) *Generalized anxiety disorder* (GAD-7) [14]; a 7-item scale, where a score of ≥10 points define generalized anxiety disorder while cut points of 5, 10, and 15 classify results into mild, moderate, and severe anxiety, and (iv) *Patient health questionnaire* (PHQ-9) [15]; a 9-item scale where the presence of symptoms during the last two weeks classifies responses into major depressive syndrome (5–9 symptoms present in more than half of the days); other depressive syndrome (2, 3, or 4 symptoms present more than half of the days); positive depressive symptoms (presence of at least one or two symptoms not fulfilling the test criteria) and negative depressive symptoms (none of the diagnostic criteria present in at least half of the days). To this study, results of GAD-7 and PHQ-9 were classified into above/below the cutoff.

The protocol was accepted by the IRB of INTA, University of Chile. Participants were invited to answer the questionnaire online, which was released via webpages in the University of Chile and Coacel (Chilean Celiac Association) websites and WhatsApp, Facebook, Twitter^®^, and Instagram. This project was approved by the IRB of INTA (INTA 260820). The study was explained in the first page and consenting to participate requested clicking a button. The survey was anonymous, and confidentiality of data was ensured.

Descriptive analyses were conducted, and graphical inspection and the Shapiro–Wilk used to assess variable’s distribution. Spearman and/or correlation assessed the association between pandemic, clinical, and mental health variables, as needed. Chi^2^ and independent t-test compared categorical and continuous variables, respectively. Univariate analysis by unadjusted logistic regression assessed the association between variables of perception of pandemic effects and the CDAT scores. All those that were statistically significant in the unadjusted regression analysis and those that might convey important information were entered into the multivariate logistic regression model. The adjusted model included adherence to GFD as dependent variables and age, sex, education, geographical macrozone, diagnosis, declared wheat consumption, cost of gluten-free foods, and GAD-7 and PHQ-9 scores as independent variables. Significance level was set at *p* < 0.05. Analyses were performed using STATA 13.1 software (Stata Corp., College Station, TX, USA) and GraphPad Prism 9 (San Diego, CA, USA) and Excel (Microsoft).

## 3. Results

### 3.1. Study Population

Of 360 responses obtained, 331 entered analysis. General characteristics of the study group appear in Table 1; 93% were women, 47% were 18–35 years of age, and 16% were older than 50 years; 70% declared university and/or post-graduate studies; 14.8% had lost their jobs; and 45.9% received public health care. Since Chile is a ≈6000 km long strip of land, with clear-cut differences in geographical characteristics and with strong people concentration in the mid macrozone, results were first analyzed by geographical origin of responders (Table 1). No significant differences were detected. Participants reported the following diagnoses: 60.4% CD, 29.3% non-celiac gluten sensitivity (NCGS), 3.2% wheat allergy (WA), and 7.3% followed GFD as a healthy feeding option or fashion, without a diagnosis that justified a restrictive diet; additional diagnoses were reported by 48%, (22.1% of which were autoimmune conditions) (Table 2). No significant differences were detected by diagnosis. Thus, results are presented as one group.

### 3.2. Perceptions of Pandemic General and Clinical Effects

Only 16% declared not to worry because of the pandemic and 37.7% worried very much. Only 14.8% of responders lost their jobs, but 84.3% experienced shortage and higher cost of food, 73% considered that they had no higher risk of infection because of their condition, and 87% declared to be worried because of shortage and higher prices of safe foods. During the last four months, 29% declared their symptoms increased, 15.7% gained weight, and 7.3% lost some, 32.6% felt no need to consult, and 36.6% failed to obtain medical help. Responders declared they increased foods preparation at home (52.3%) and ingredients were changed to some not customarily used but considered safe; 53.8% consumed gluten-containing foods during the pandemic period. Responses of persons on GFD for less than 1 year did not differ from those of participants on the diet for longer periods.

### 3.3. Health Eating Index (HEI)

The HEI mean score for the study group was 65.6 ± 13.3 points, which classifies in the intermediate level in the HEI scale i.e., “needs improvement” of the feeding patterns. While 15.8% maintained a healthy diet, 71.3% were classified in the intermediate level and in 11.5%, the diet was not healthy.

### 3.4. Adherence

Given the relevance of GFD in treating GRD, adherence was assessed by both the responder’s perception and by CDAT score (Table 3). While 49.9% perceived that their adherence worsened during pandemic, in 44.4% had a CDAT score that classified them in the “bad adherence” category (≥13 points). Positive Spearman correlation consistently found the self-perception of adherence to GFD and CDAT score positively correlated with most dietary and clinical variables measured.

### 3.5. Mental Health

In total, 28% of the participants obtained scores above 10 (cut off for anxiety) in GAD-7 and 40% obtained above score 10 (cut off for depression) in PHQ-9; 23% were above the cutoff in both tests (Table 1). Chi^2^ analyses revealed that positive score in GAD-7 was significantly associated with being worried for the pandemic, risk of infection with COVID-19, and shortage of safe foods (all *p* < 0.001). Spearman and Pearson correlations revealed some diverse but inconsistent correlations with the general and specific variables assessed (Table S1); instead, adherence-related variables showed that the poorer the perception of adherence, the stronger the perception of anxiety or depression or both (*p* < 0.0000, Table 4). Analysis of depression showed that health insurance, perception of dietary quality deterioration, increased prices, wheat consumption, self-perception of adherence, presence of current symptoms, cooking at home, and need to consult were significantly associated with positive PHQ9 scores (all *p* < 0.004, Table 4). Associations of mental health indicators and adherence to GFD were strong, both by perception and by CDAT score (Table 4).

### 3.6. Logistic Regression

Given the results of adherence and mental health, logistic regression models using perception of self-adherence and CDAT as dependent variables were calculated. Both models were concordant; the one using adherence (CDAT score) to GFD as a dependent variable and, age, sex, education, geographical macrozone, diagnosis, declared wheat consumption, cost of gluten-free foods, GAD-7 and PHQ-9 scores as independent variables, shows that in participants stating that the pandemic affected their GFD, the likelihood of showing poor adherence by CDAT was 2.3 times higher (OR 2.3, IC 1.3–4.2, *p* < 0.004). Responders that suffered depressive symptoms were 1.3 times more likely to have poor adherence (OR 1.3, IC 1.2–1.4, *p* < 0.000). Older age (OR 0.97, IC 0.94–0.99, *p* < 0.038) and living in the south macrozone (OR 0.5; IC 0.27–0.97, *p* < 0.041) were associated with lower CDAT score, indicating better adherence.

## 4. Discussion

Results show that after several months of living in COVID-19 pandemic conditions, relevant effects can be indeed detected in persons with CD and other GRD maintaining GFD. Perception of significant deterioration of adherence to diet and mental health are the two most significant findings; unfortunately, because the pandemic situation is unprecedented, it is not possible to evaluate results using a formal before-and-after analysis or against comparison/control groups. Results also suggest the development of strategies to cope with the difficulties faced; it is interesting that those who follow GFD as treatment do not seem to differentiate from those who follow it as a fashion/trend that consider it a “healthy diet” [16].

### 4.1. Self-Perceptions of the Pandemic and Clinical Aspects

As previously reported [5], “very much concern” for the pandemic is high (37.7%), and only 16% feel not worried at all. That 73% of responders perceive no higher risk of infection concur with earlier publications and reports [5,17]. Loss of jobs, shortage of food, and higher cost of safe products are the most relevant factors responders identified as determinants of difficulties for adhering to GFD (x2, all *p* < 0.0001); this concurs to an FAO recent statement acknowledging shortage of food as a relevant factor influencing present general dietary quality [18]. It seems reasonable that poor adherence is associated with increasing symptoms. Yet, the exact causes for the increase remain unclear because several factors may be involved, such as the rapid deterioration of adherence to GFD, increased stress inducing unspecific gastrointestinal complaints; mild COVID-19 infection [19], altered permeability [20,21], and modifications to the microbiome that can favor local inflammation [21,22]. It was unexpected that persons declaring CD and NCGS reported the same frequency of autoimmune conditions; however, autoimmune symptoms have been already described in NCGS by other authors [23,24]. Participants developed strategies to meet their dietary needs, the proportion of patients with “unhealthy diets” was low (11.5%), and one-third (32.6%) of responders did not need to seek medical help; but at the same time, symptoms increased during the last four months, and a high proportion of patients did try to find medical help. So, since the participants who were chronic patients were used to challenges when deciding what to eat, we interpret these results as that they are certainly resilient, which agrees with Monsani’s report [25], but they definitely suffer adverse pandemic effects, and these are strong and consistent. It is intriguing that the HEI group mean score (65.7 points) observed, which classifies as “needs improvement”, does not differ from the mean score described for general population in the previous national survey (2017) [26]. Lack of data in persons on GFD prior to the pandemic impedes further analysis of these results. Anyway, it is interesting that results of HEI and CDAT (validated tests) are concordant with those obtained when asking for perceptions. It was unexpected that being on GFD for a shorter time made no difference when compared with persons on GFD for many years.

### 4.2. Mental Health

Both GAD-7 and PHQ-9 were positively related with variables representing the pandemic, dietary, and clinical characteristics; however, relations with positive depression markers were more numerous and consistently significant. Correlation analysis against variables related to diet and clinical aspects showed weak associations (Table S1), and instead, association to variables assessing adherence to GFD was strong (Table 4). The several analyses performed to test the strength of the associations (Table 4) confirmed the relationship between mental health scores and adherence, both by perception and CDAT scores, leaving these variables as the main pandemic effects in daily life of people suffering GRD. 

Experiences during EBOLA and SARS pandemics support the idea that mental health could be specially affected during the current COVID-19 pandemic [27,28]. Limitations to free moving, uncertainty, and fear facing the advances of viral infections, lack of physical activity, technological capacities required to access food, remote working, and confinement are fundamental factors shaping the mental health deterioration of the general population during the current pandemic [29]. It is difficult to discuss present results because the available data are scarce and incomplete. During the pandemic, the frequency of depression and anxiety in the general population was high in China (48% and 23%, respectively) [30] and in Hong Kong (20% and 14%, respectively) [29]; and in the USA, depression increased three times (prevalence 28%) [31]. As for CD, a recent systematic review and meta-analysis assessing celiac patients in non-pandemic conditions [32] reported that depression (OR 2.17, 95% CI 2.17–11.15, *p* < 0.0001) and anxiety (OR 6.03, 95% CI 2.22–16.35, *p* < 0.0001) were higher in celiac patients compared with general population. In Chile, the last National Health Survey (2017) [33] showed the prevalence of depression at 6.2% in general population. Our current results are higher than these figures, but they do not clarify to what extent the differences are due to the presence of disease or the pandemic. A limitation to this study is that the group assessed was formed mainly by women, making it difficult to explain whether sex is a relevant variable in the analysis; anyway, depression was twice more frequent among women than in men (10% vs. 2%). Although logistic regression shows a strong relationship between adherence, being worried by the pandemic and mental health, it is unclear how these factors interact between them. Older age and living in smaller, uncrowded cities appear as the only protective factors detected in this study.

Limitations to this study are clear but mostly unavoidable, forcing it to remain descriptive. Since the COVID-19 pandemic is unprecedented and started abruptly, data prior to the pandemic are not available in the study group and comparisons of before and after the pandemic are not possible. The fact that it is still ongoing impedes having control/comparison groups, which is a situation that will change in time, enabling longitudinal analyses. In addition, due to many persons maintaining anonymity, we did not obtain clinical data. Despite this, the results described are relevant because no matter the exact causes of the effects described, and no matter how they compare to situations prior to the COVID-19 pandemic, depression and faulty GFD (mandatory for treatment) appear to be affecting a high proportion of participants, suggesting that support measures aimed at these aspects would help improve the health condition of people that maintain GFD. Finally, it is also important to emphasize that comparisons between this and other studies available should be cautious, because not only cultural aspects but also the conditions and timing of data collection are most variable. Further studies are indeed necessary to better understand the problems derived from the COVID-19 pandemic in persons that must follow restrictive diets such as GFD.

## 5. Conclusions

We conclude that after nearly a year living under COVID-19 pandemic conditions, the deterioration of dietary treatment and mental health of individuals suffering GRD appear as main effects of the COVID-19 pandemic. Loss of jobs, shortage of food, prices increase, poor access to safe foods, increase of symptoms, and poor availability of medical help are the relevant difficulties identified by individuals on GFD. Although the group assessed developed some strategies to cope with dietary pandemic problems, these fail to guarantee good adherence to treatment (GFD), maintain a healthy diet, and preserve good mental health. Depression represents the most relevant alteration in mental health assessment. Results of this study allow better understanding the health-related pandemic effects in people following restrictive diets, suggesting that improving the capacity to maintain adherence to diet and provide mental health support are two main areas that may help focusing interventions for this group within the population.

## Figures and Tables

**Table 1:** General characteristics, diagnoses declared, and mean group scores in dietary and mental health tests by geographic zone of origin of participants.

Variable	Northern Zone (*n* = 26)	Central Zone (*n* = 259)	Southern Zone (*n* = 46)	Total (*n* = 331)	*p* Value
Age (years)	42.1 ± 11.1	37.3 ± 11.9	36.8 ± 11.3		0.13 **
Gender (F), *n* (%)	24 (92.3)	245 (94.6)	41 (89.1)	310 (93.7)	0.93
Urban housing, *n* (%)	24 (92.3)	251 (96.9)	38 (82.6)	313 (94.6)	0.25
Education					0.86 **
Primary-high school, *n* (%)	4 (15.4)	53 (20.5)	9 (2.0)		
University, *n* (%)	17 (65.4)	152 (58.7)	25 (54.4)		
Postgraduate studies	5 (19.2)	54 (20.8)	12 (26.1)		
Public health insurance, *n* (%)	13 (50)	111 (42.9)	26 (56.5)	149 (45.0)	0.27
Diagnoses declared, *n* (%)					0.291 **
Wheat allergy, *n* (%)	2 (0.8)	8 (3.1)	0	10 (3.2)	
Celiac disease, *n* (%)	15 (57.7)	162 (62.6)	23 (50.0)	200 (60.4)	
NCG/WS, *n* (%)	6 (23.1)	72 (27.8)	19 (41.30)	97 (29.3)	
None, *n* (%)	3 (11.5)	17 (6.6)	4 (8.7)	24 (7.3)	
CDAT, score (mean ±SD)	14.6 ± 5.2	14.1 ± 4.7	13.1 ± 4.4		0.30 *
HEI score (mean ±SD)	68.4 ± 9.5	65.2 ± 13.5	66.3 ± 14.2		0.47 **
GAD-7 score (mean-range)	6 (3–11)	7 (4–12)	7(4–13)		0.88 **
PHQ-9 score (mean-range)	9 (5–13)	9 (4–14)	7 (5–13)		0.81 **

Data as mean ± SD. * Kruskal-Wallis Test; ** One-way ANOVA test; Fisher’s exact; NCG-WS: non celiac gluten/wheat sensitivity. CDAT: Celiac Dietary Adherence Test; HEI: Healthy Eating Index; GAD-7: Generalized anxiety disorder test; PHQ-9: Patient health questionnaire.

**Table 2:** CDAT score, non-celiac autoimmune diseases declared, and years on GFD by declared diagnoses.

	CD*	NC/WGS**	WA***	No Diagnosis Declared ****
*n* (%)	200 (60%)	97 (29.3%)	10 (3%)	24 (7.2%)
CDAT score < 13 ^†^, *n* (%)	93 (46.5%)	37 (38.1%)	7 (70%)	10 (4.2%)
Additional autoimmune disorders declared	48 (24%)	21 (21.7%)	1 (10%)	3 (12.5%)
Duration of gluten-free diet				
Up to 1 year (*n*, %)	2 (1)	0	0	0
1–5 years (*n*, %)	119 (59.5)	91 (93.8)	5 (50)	23 (95.8)
>5 years (*n*, %)	79 (39.5)	6 (6.2)	5 (50)	1 (4.2)

CD*: celiac disease; NC/WGS** non-celiac gluten/wheat sensitivity; WA***: wheat allergy. ****: persons that follow GFD as healthy feeding option or follow a fashion/trend. ^†^ CDAT score < 13 points indicate bad adherence to GFD.

**Table 3:** Self-perception of COVID-19 pandemic effects on gluten-free diet and celiac disease test adherence (CDAT).

	Adherence Self-Perception of		Adherence CDAT			
Survey Questions	“Good”	“Bad”	Poor Adherence	Good Adherence	*p* Value *	
**Has the increase in food prices in general affected the quality of your diet? (yes) *n* (%)**	207 (62)	124 (37.4)	129 (38.7)	78 (23.6)	0.0001	0.0001
**How many wheat-containing foods do you include in your diet per week? *n* (%)**						
*1 Four or more times*	6 (3.6)	1 (0.5)	7 (3.8)	0 (0)	0.0001	0.0001
*2 Two or three times*	19 (11.6)	5 (2.9)	22 (12.1)	2 (1.3)		
*3 None*	115 (70.5)	153 (91)	125 (69)	143 (95.3)		
*4 Once*	23 (14.1)	9 (5.3)	27 (14.9)	5 (3.3)		
**How do you consider your diet? *n* (%)**						
*1 Excellent*	20 (13.2)	55 (32.7)	22 (12.1)	53 (35.3)	0.0001	0.0001
*2 Fairly good*	79 (48.4)	104 (61.9)	94 (51.9)	89(59.3)		
*3 Not so good*	58 (35.5)	9 (5.3)	60 (33.1)	7 (4.6)		
*4 Badly done*	6 (3.6)	0 (0)	5 (2.7)	1 (0.6)		
**Does the possibility of shortage of safe gluten-free foods during the pandemic worries you? *n* (%)**						
*1 Yes*	135 (83.8)	80 (47.6)	125 (69)	90 (60)	0.0001	0.012
*2 Slightly*	22 (13.5)	51 (30.3)	37 (20.4)	36 (24)		
*3 No*	6 (3.6)	37 (22)	19 (10.5)	24 (16)		
**Does maintaining social distancing when going to the supermarket or crowed places worries you? *n* (%)**						
*1 Yes*	112 (68.7)	89 (52.9)	122 (67.4)	79 (52.6)	0.004	0.022
*2 A little*	34 (20.8)	43 (25.5)	36 (19.8)	41 (27.3)		
*3 Not*	17 (10.4)	36 (21.4)	23 (12.7)	30 (20)		
**Does the COVID-19 pandemic worry you? *n* (%)**						
*1 Very much concerned*	75 (46)	79 (47)	78 (43.1)	76 (50.6)		
*2 A lot*	74 (45.3)	47(27.9)	79 (43.6)	42 (28)	0.0001	0.012
*3 A little*	1 (0.6)	1 (0.5)	1 (0.6)	1 (0.6)		
*4 No*	13 (7.9)	41(24.4)	23 (12.7)	31 (20.6)		
**Do you feel that you have higher risk of infection with COVID-19 due to your diagnosis? *n* (%)**						
*1 Very much concerned*	36 (22)	14 (8.3)	31 (18.1)	19 (12.6)		
*2 A lot*	30 (18.4)	10(7.2)	29 (16)	11 (7.3)	0.0001	0.03
*3 A little*	55 (33.7)	94 (55.9)	78 (43.1)	71 (47.3)		
*4 No*	42 (25.7)	50(29.7)	43 (23.7)	49 (32.6)		
**Are you getting the gluten-free foods that you regularly obtained before the pandemic? *n* (%)**						
*1 I have problems, but I can get them*	80 (50)	81(48.2)	82 (45.3)	79 (52.6)		
*2 It has been most difficult to get them*	66 (40.4)	18(10.7)	58 (32)	26 (17.3)	0.0001	0.002
*3 I have not been able to get them*	4 (2.4)	0 (0)	4 (2.2)	0 (0)		
*4 I have had no problems.*	13 (7.9)	69 (41)	37 (20.4)	45 (30)		
**Have the prices of gluten-free foods increased? (yes) *n* (%)**	141 (42.5)	126 (38.1)	152 (45.9)	115 (34.7)	0.008	0.12
**Cooking gluten-free foods at home during the pandemic *n* (%)**						
*1 Increase*	81 (49.6)	92 (54.7)	97 (53.5)	76 (50.6)	0.0001	0.001
*2 Decreased*	23 (14,1)	1(0.5)	21 (11.6)	3 (2)		
*3 Has not changed*	59 (36.1)	75 (44.6)	63 (34.8)	71 (47.3)		

* (perception of adherence).

**Table 4:** Associations of positive anxiety (GAD-7) and depression (PHQ-9) scores with pandemic effects and adherence to diet (by perception and CDAT).

	GAD-7			PHQ-9			Positive Score in Both Tests		
Variable	Chi^2^ (*p*)	TE (r)	Spear (Pos; *p*) *	Chi^2^ (*p*)	TE (r)	Spear (Pos; *p*)	Chi^2^ (*p*)	TE (r)	Spear (Pos; *p*)
Higher cost of food	<0.0001	0.2154	pos < 0.001	<0.0001	0.3469	pos < 0.001	<0.0001	0.1961	pos < 0.001
“Pandemic Affected Adherence”	<0.0001	0.2408	pos < 0.001	<0.0001	0.287	pos < 0.001	<0.0001	0.2143	pos < 0.001
Consumption of Gluten	0.014	0.1747	pos < 0.003	0.0017	0.1978	pos < 0.001	0.0091	0.1653	pos 0.004
Adherence by perception	0.0001	0.203	pos < 0.001	<0.0001	0.2755	pos < 0.001	<0.0001	0.2469	pos < 0.001
CDAT	<0.0001	0.3981	pos < 0.001	<0.0001	0.4682	pos < 0.001	<0.0001	0.3914	pos < 0.001

* Pos; *p* = positive correlation; *p* value.

## Supplementary Materials

The following is available online at https://www.mdpi.com/article/10.3390/nu13061822/s1, Table S1: Associations of anxiety (GAD-7) and depression (PHQ-9) with pandemic effects.
