# Supplementary material for: Pandemic Effects and Gluten-Free Diet: An Adherence and Mental Health Problem
Source: Nutrients. 2021 May 27;13(6):1822. doi: 10.3390/nu13061822 (PMC8229361; doi:10.3390/nu13061822)
Supplement: Supplementary file 1 [file nutrients-13-01822-s001.zip › nutrients-1191452-supplementary.pdf]

Table S1. Associations of anxiety (GAD-7) and depression (PHQ-9) with pandemic effects.

Variable	**GAD-7** chi² (p)	**PHQ-9** chi² (p)	**Both scores positive** chi² (p)
Geographical origin	0,85	0,882	0,7351
Housing conditions	1	0,3659	0,5398
Education level	0,67	0,1952	0,6952
Health insurance	0,24	0,0043	0,207
Shortage of foods	0,067	0,0002	0,0562
Isolation	0,0091	0,0028	0,0326
Worrying about Pandemic	0,0006	0,0016	0,0038
Worrying for risk of infection	0,009	<0,0001	0,0034
Difficulties to obtain gluten-free food	0,0001	<0,0001	<0,0001
Higher cost of gluten-free food	0,2215	0,0089	0,3525
Frequency of home cooking	0,1883	0,0034	0,0906
Symptoms during last four months	<0,0001	<0,0001	0,0001
Need to consult during last 4-6 months	0,0577	0,0026	0,048
Past occupation	0,0256	0,1262	0,1003
Present occupation	0,1368	0,0145	0,1532
